# Comparative Metabolomic and Transcriptomic Studies Reveal Key Metabolism Pathways Contributing to Freezing Tolerance Under Cold Stress in Kiwifruit

**DOI:** 10.3389/fpls.2021.628969

**Published:** 2021-06-01

**Authors:** Shihang Sun, Jinbao Fang, Miaomiao Lin, Chungen Hu, Xiujuan Qi, Jinyong Chen, Yunpeng Zhong, Abid Muhammad, Zhi Li, Yukuo Li

**Affiliations:** ^1^Key Laboratory for Fruit Tree Growth, Development and Quality Control, Zhengzhou Fruit Research Institute, Chinese Academy of Agricultural Sciences, Zhengzhou, China; ^2^Key Laboratory of Horticultural Plant Biology (Ministry of Education), College of Horticulture and Forestry Science, Huazhong Agricultural University, Wuhan, China

**Keywords:** kiwifruit, freezing tolerance, RNA-Seq, metabolome, cold stress, UPLC-ESI-MS/MS

## Abstract

Cold stress poses a serious treat to cultivated kiwifruit since this plant generally has a weak ability to tolerate freezing tolerance temperatures. Surprisingly, however, the underlying mechanism of kiwifruit’s freezing tolerance remains largely unexplored and unknown, especially regarding the key pathways involved in conferring this key tolerance trait. Here, we studied the metabolome and transcriptome profiles of the freezing-tolerant genotype KL (*Actinidia arguta*) and freezing-sensitive genotype RB (*A. arguta*), to identify the main pathways and important metabolites related to their freezing tolerance. A total of 565 metabolites were detected by a wide-targeting metabolomics method. Under (−25°C) cold stress, KEGG (Kyoto Encyclopedia of Genes and Genomes) pathway annotations showed that the flavonoid metabolic pathways were specifically upregulated in KL, which increased its ability to scavenge for reactive oxygen species (ROS). The transcriptome changes identified in KL were accompanied by the specific upregulation of a codeinone reductase gene, a chalcone isomerase gene, and an anthocyanin 5-aromatic acyltransferase gene. Nucleotides metabolism and phenolic acids metabolism pathways were specifically upregulated in RB, which indicated that RB had a higher energy metabolism and weaker dormancy ability. Since the LPCs (LysoPC), LPEs (LysoPE) and free fatty acids were accumulated simultaneously in both genotypes, these could serve as biomarkers of cold-induced frost damages. These key metabolism components evidently participated in the regulation of freezing tolerance of both kiwifruit genotypes. In conclusion, the results of this study demonstrated the inherent differences in the composition and activity of metabolites between KL and RB under cold stress conditions.

## Introduction

Low temperature stress is one of the main factors that restricts the development and growth of plants, limits their distribution and causes significant losses in yield (Peng et al., 2015). Enhancing the cold tolerance of fruit trees via breeding is a prerequisite to better cope with frequent extremely low temperatures (Xiaoming et al., 2019). A plant’s cold tolerance mechanism can be divided into two parts: its chilling tolerance and the freezing tolerance (FT) (Sun et al., 2020). Chilling tolerance reflects the ability of a plant to respond to 0–15°C growing conditions (Chen et al., 2020), whereas FT is its ability to respond to subzero temperatures (Zhang et al., 2020). While the chilling tolerance mechanism has been elucidated at transcriptional and metabolic level (Jingyu et al., 2016), the mechanism of FT still remains largely unknown.

Moreover, the FT mechanism differs between annual plant and perennial plant species. For annuals, such as *Arabidopsis*, FT is a multigenic and quantitative trait that relies more on changes in metabolites levels. Many metabolites linked to the FT trait, such as osmolytes (proline, betaine, putrescine) and reactive oxygen species (ROS)-scavenging systems (catalase, peroxidase, superoxide dismutase, lignan and ascorbic acid), are known to have an important role in maintaining the survival of annual plants under cold conditions (Li et al., 2017; Ding et al., 2018; Yao et al., 2020). FT mainly derives from timely osmotic adjustments and an ability to scavenge reactive oxygen species (ROS). Unlike for annuals, the studies of FT in perennial plants have mainly focused on the seedling stage rather than adults; however, the responses to cold stress in seedlings likely differ between them in actual field situations (Chai et al., 2019). To resolve this imbalance, we designed an experiment using 3-year-old (i.e., adult stage) kiwifruit which had already entered the dormant stage, thus offering an identical situation to that in a field setting.

Recently, metabolic responses to cold stress in plants have garnered more attention, since numerous metabolites are considered to play vital roles in FT (Singh et al., 2020). In the process of adjusting to cold stress, the plant produces secondary metabolites as a kind of excretory compound, and these metabolites are the real driver of plant stress responses (Lee et al., 2019). Accordingly, changes in metabolites contents are considered to be ultimate response of plants to abiotic stresses (Ma et al., 2016). The metabolomics technique is a powerful tool for detecting and analyzing metabolites in plants under cold stress, and this approach can shed new light on the their response mechanisms to a low-temperature environment (Clemente-Moreno et al., 2020). For example, the accumulation of sugars and amino acids can occur in this cold response according to a recent metabolome study (Hannah et al., 2006). Cold stress can also influence the nucleic acid and protein stability, enzyme activity, and cytoskeleton structure (Cook et al., 2004). Slowed or even stunted plant growth due to cold stress will generally decrease the amplitude of energy utilization, by causing the formation of ROS (Dreyer and Dietz, 2018), and a low temperature induces the accumulation of anthocyanin to scavenge ROS (An et al., 2018). It has been reported as well that polyamines are involved in potato’s cold response to eliminate ROS (Kou et al., 2018). Plants produce osmolytes–i.e., proline, betaine, raffinose, trehalose and inositol–to deal with the low temperature stress, since these metabolites under cold conditions have protective functions toward alleviating damage from freezing (Zhao et al., 2019). Similarly, plants accumulate suberin and lignin to adjust themselves to cold stress and reduce its adverse effects (Jian et al., 2020). The fluidity of membrane composition plays an central role in temperature perception, which reduces to form a solid gel capable of sensing cold stress (Gilad et al., 2018; Yang et al., 2019), while changes in the content of soluble sugars may play a key role in cold signaling transduction by regulating the expression of cold-responsive genes (Krasensky and Jonak, 2012). Besides metabolomics, high-throughput sequencing techniques like transcriptomics have been widely used to explore the problem of coping with abiotic stress in a wide range of plants (Bahrman et al., 2019). The ICE–CBF–COR regulation network response to cold stress was discovered using RNA-Seq technology, in which cold stress induces the expression of transcriptional factors, including AP2-domain protein CBFs, which activate the expression of various downstream cold responsive (COR) genes (Stockinger et al., 1997; Wanqian et al., 2018). The ICE (bHLH transcription factors) controls the *CBF* genes via sumoylation and polyubiquitylation that is mediated by SUMO-E3-ligase SIZ1 and ubiquitin-E3-ligase HOS1, respectively (Zhang et al., 2016). In this way, by combining profiling of the metabolome and transcriptome, a more extensive and more comprehensive understanding of metabolites and genes co-regulation of cold stress was elucidated.

Kiwifruit (*Actinidia*) is an economic plant domesticated in recent decades that is mainly distributed in subtropical and temperate regions (Yukuo et al., 2018). In these climatic zones, the winter always entails extremely low temperatures, which makes the kiwifruit vulnerable to freezing damage during the overwintering period. This freezing injury results in reduced kiwifruit yield and it can also detrimentally affects the survival of the whole plant. *Actinidia arguta* is a special species that has a large range, from northeast to south of China (Lin et al., 2020). Two contrasting genotypes in *A. arguta* have been identified, these being KL and RB. While former grows naturally in higher latitude regions and exhibits a relatively strong FT, the latter typically occurs in lower latitude areas and its FT is weak. The divergence of the two kiwifruit genotypes in terms of their FT trait renders them an ideal model for studying how plants respond to a low-temperature environment.

Here, a naturally existing experimental system—two kiwifruit genotypes with significantly different freezing tolerance ability—was used to study the mechanism of FT in kiwifruit. We conducted comparative metabolomic and transcriptomic analyses at a series of time points to identify global dynamic changes and uncover the metabolic models underlying their cold response. These results improve our understanding of kiwifruit’s FT mechanism, and provide useful information for enhancing the FT in kiwifruit.

## Materials and Methods

### Plant Material and Cold Stress Treatments

To discern FT mechanism, we used two kiwifruit (*A. arguta*) genotypes known to differ greatly in their tolerance of freezing: the high-freezing tolerance genotype KL and the low freezing tolerance genotype RB. The mature (3-year-old) plants of each genotype (individually planted in a 3-L-pot) were placed in a field for 3 years at Zhengzhou fruit research institute (113°E, 34°N). Zhengzhou city has a temperate climate with short-day in winter and temperature reaches to −15°C. All the shoots were collected in early January 2020, then the detached shoots were placed in a −25°C freezer chamber without light (Suzhou Zhihe Instrument Factory, China), when three biological replicates of shoots for metabolic profiling and for RNA sequencing (RNA-Seq) were collected randomly from all plants of each genotype at time points of 0 h, 1 h, 4 h, and 7 h under a −25°C treatment. At the same times, shoots were collected to assess their vegetative budbreak (VB), relative electrolyte leakage (REL) and other physiological parameters. The collected samples were immediately frozen in liquid nitrogen and stored at −80°C until their RNA and metabolite extractions.

### Relative Electrolyte Leakage Assay

After receiving the low temperature treatment, the kiwifruit shoots were cut into 1−2-mm thick slices. Then 0.2 g of these slices per sample per genotype were incubated in 30 ml of double-distilled water (ddH2O) for 2 h, with shaking at 200 rpm at 25°C. Initial electrolyte leakage (C1) was measured using a digital conductivity meter (DDS−307, Rex, China), with their second electrolyte leakage (C2) likewise measured after the samples had been boiled at 100°C for 30 min, then cooled down at 25°C with 30 min of shaking. The REL was calculated this way:

(1) $$REL\left(\%\right)={\left(C1/C2\right)}^{*}100\%$$

The LT50 (i.e., the semi-lethal temperature at which REL reaches 50%) was calculated by fitting a logistic sigmoid function to the REL values:

(2) $$y=k/\left(1+ae^{-\mathrm{bx}}\right)$$

where x is given treatment temperature; y is the REL value; k is the maximum value when x approaches infinity; ‘a’ and ‘b’ are the estimated equation parameters.

### Evaluation of Vegetative Budbreak

To examine the differences in freezing tolerance, the VB was evaluated at each collection time point in three shoots (length ≥ 20 cm) having five or more vegetative buds per shoot (at least 15 buds in total) that were pruned from both genotypes (Zhao et al., 2020). For budbreak induction, three shoots were placed in 100 ml tissue-culture bottles containing ddH_2_O, then incubated in a controlled-climate chamber at 24 ± 1.0°C under a 16-h/8-h photoperiod, to induce budbreak. The ddH_2_O in the bottles was changed every 2 to 3 days. The VB was determined for each collection time points after the samples spent 20 days in the controlled chamber. The statistical differences in VB were tested with ANOVAs implemented in SPSS (IBM Corp., Armonk, NY, United States).

### Measurement of Anti−O_2_^.–^ Capacity and Contents of Procyanidin, Flavonoid

The anti−O_2_^.–^ capacity, the contents of procyanidin, flavonoid were measured using corresponding assay kits according to the manufacturer’s instructions (Nanjing Jiancheng Bioengineering Institute, China). The principle of anti−O_2_^.–^ determination was based on the reaction of ‘xanthine’-‘xanthine oxidase’-‘gress chromogenic agent’ and anti−O_2_^.–^ was determined by detecting the absorption value at 560 nm. The procyanidin was determined by reacting it with vanillin to produce the colored compounds, the absorption value was determined at 500 nm. The flavonoid determination was done by reacting it with aluminum ions untill produced the red compounds, the absorption value was measured at 502 nm. The activity of anti−O_2_^.–^ was indicated as unit (U) per g fresh weight. The contents of procyanidin and flavonoid were shown as mg per g fresh weight.

### Metabolomic Profiling and Statistical Analysis

The freeze-dried shoots were crushed in a mixer mill (MM 400, Retsch, Germany) with zirconia beads for 1.5 min at a frequency of 30 Hz. From the ensuring power, 100 mg was weighted and extracted overnight at 4°C with 0.6 ml of 70% aqueous methanol. Following centrifugation at 10 000 × *g* for 10 min, the extracts were absorbed and filtrated before their UPLC-MS/MS (ultra performance liquid chromatography/tandem mass spectrometry) analysis. The sample extracts were examined in an UPLC-ESI-MS/MS system (UPLC, Shim-pack UFLC SHIMADZU CBM30A system; MS, Applied Biosystems 4500 Q TRAP). For its analytical conditions, a UPLC Agilent SB-C18 column (1.8 μm, 2.1 mm^∗^100 mm) was used with a mobile phase consisting of solvent A (pure water with 0.1% formic acid) and solvent B (acetonitrile). Sample measurements were performed with a gradient program whose starting conditions was 95% A, 5% B; within 9 min, a linear gradient inversion to 5% A, 95% B was programmed, and this latter composition held for 1 min. Then, a composition of 95% A, 5.0% B was adjusted within 1.1 min and kept for 2.9 min. The column oven was set to 40°C and the injection volume was 4 μl. The effluent was alternatively connected to an ESI-triple quadrupole-linear ion trap (QTRAP)-MS.

LIT and triple quadrupole (QQQ) scans were acquired on a triple quadrupole-linear ion trap mass spectrometer (Q TRAP [API 4500 Q TRAP UPLC/MS/MS System]) equipped with an ESI Turbo Ion-Spray interface, operating in the positive and negative ion mode and controlled by Analyst 1.6.3 software (AB Sciex, Washington, DC, United States). The ESI source operation parameters were as follows: ion source, turbo spray, source temperature of 550°C, ion spray voltage (IS) of 5500 V (positive ion mode)/−4500 V (negative ion mode), for which the ion source gas I (GSI), gas II(GSII), and curtain gas (CUR) were set at 50, 60, and 30.0 psi, respectively; the collision gas(CAD) was set to high. Instrument tuning and mass calibration were performed with 10 and 100 μmol/L polypropylene glycol solutions in the QQQ and LIT modes, respectively. QQQ scans were acquired as MRM experiments with a collision gas (nitrogen) set to 5 psi. The DP and CE for individual MRM transitions was done with further DP and CE optimization. A specific set of MRM transitions were monitored for each period according to the metabolites eluted within this period. The qualitative identification of metabolites was obtained from the metabolite information available on public database (MassBank, KNAPSAcK, and METLIN) and private MVDB database (Wuhan Metware Biotechnology Corporation, China).

Unsupervised PCA (principal component analysis) was performed using the statistics function ‘prcomp’ within R^1^. The data was unit variance-scaled before conducting the unsupervised PCA. The HCA (hierarchical cluster analysis) results of samples and metabolites were presented as heat maps with dendrograms, while Pearson correlation coefficients (PCC) between samples were calculated by the ‘cor’ function in R and presented as heat maps only. Both HCA and PCC were carried out by R package ‘pheatmap’. For the HCA, the normalized signal intensities of metabolites (unit variance scaling) were visualized as a color spectrum. Significantly regulated metabolites between groups were designated as those with a VIP (variable importance in projection) ≥ 1 and absolute Log2FC (fold change) ≥ 1. The VIP values were extracted from OPLS-DA (Orthogonal Partial Least Squares Discrimination Analysis) results; the latter included score plots and permutation plots, as generated by the R package ‘MetaboAnalystR’. The data was log transform (log2) and mean-centered before applying the OPLS-DA. To avoid overfitting, a permutation test (*n* = 200 resamplings) was performed. Identified metabolites were annotated using the KEGG Compound database^2^, and latter mapped to the KEGG Pathway database^3^. Those mapped pathways with significantly regulated metabolites were then fed into MSEA (metabolite sets enrichment analysis); their statistical significance was determined by *p*-values from hypergeometric tests.

### RNA-Seq and Statistical Analysis

Total RNA was isolated from the shoots (100 mg per sample) with a plant total RNA purification kit (Waryoung, Beijing, China) by following the manufacturer’s instructions. Samples from the four collection time points were subjected to RNA-Seq. In all, 24 samples were used for this RNA-Seq (2 cultivars × 4 collection times × 3 biological replicates). RNA quality was determined in a NanoDrop 1000 spectrophotometer, after which the mRNA was extracted using dynabeads oligo (dT) and a fragmentation buffer. Double-stranded cDNAs were synthesized using reverse-transcriptase and random hexamer primers, and the cDNAs’ fragments were purified with a QIA quick PCR extraction kit. These purified fragments were washed with an EB buffer for end reparation of the poly (A) addition and then ligated to sequencing adapters. Following agarose gel electrophoresis and extraction of cDNAs from the gels, the cDNAs’ fragments were purified and enriched by PCR to construct the final library of cDNAs. This library was then sequenced on the Illumina sequencing platform (Illumina Hi-SeqTM 2500) by using paired-end technology.

After removing the adapter and any low-quality sequences, the clean reads were obtained and mapped to the transcripts assembled by Trinity. The mapping were done using bowtie2 (RSEM). The FPKM (fragments per kilobase of exon per million mapped fragments) was used to calculate gene expression levels, and differentially expressed genes (DEGs) were screened by Cuffdiff according to these two criteria: having (i) a false discovery rate (FDR) corrected *P*-value < 0.05 and (ii) a | log2(fold change) | ≥ 1. Based on the ‘blastp’ analysis, the annotated genes in kiwifruit were screened for further analysis. Finally, all sequencing data obtained in this study has been uploaded to NCBI.

## Results

### Freezing Tolerance Assessment and Low Temperature Treatment of the KL and RB Genotypes

**FIGURE 1:**
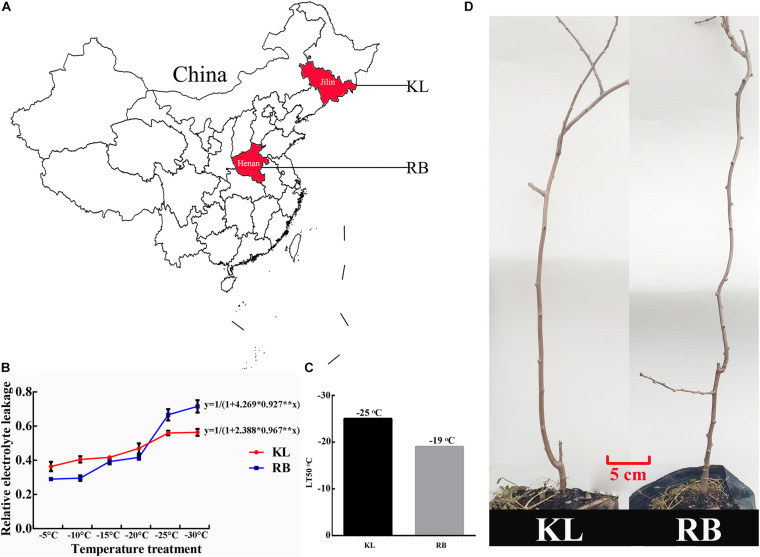
Geographic distribution and freezing tolerance of the *A. arguta* KL and RB plants. **(A)** Both genotypes were derived from a different region; the KL came from Jilin province (125°E, 44°N), the RB was from Henan province (113°E, 34°N). **(B)** The logistic method was used to calculate the LT50 of each genotype. **(C)** KL and RB exhibited high (−25°C) and low (−19°C) freezing tolerance, respectively. **(D)** Three-year-old plants of both genotypes were used in this study.

The KL came from Jilin province (125°E, 44°N), and the RB was from Henan province (113°E, 34°N) (Figure 1A). The lowest winter temperature is about −30°C in Jilin province, while it is about −15°C in Henan province (data from http://data.cma.cn/). Their LT50 values of dormant shoots were −25°C (KL) and −19°C (RB), respectively (Figures 1B–D), which indicated that KL and RB were respectively tolerant of and sensitive to cold stress. The distinct FT trait between the two genotypes makes them an excellent model for investigating the underlying mechanisms of kiwifruit’ FT.

**FIGURE 2:**
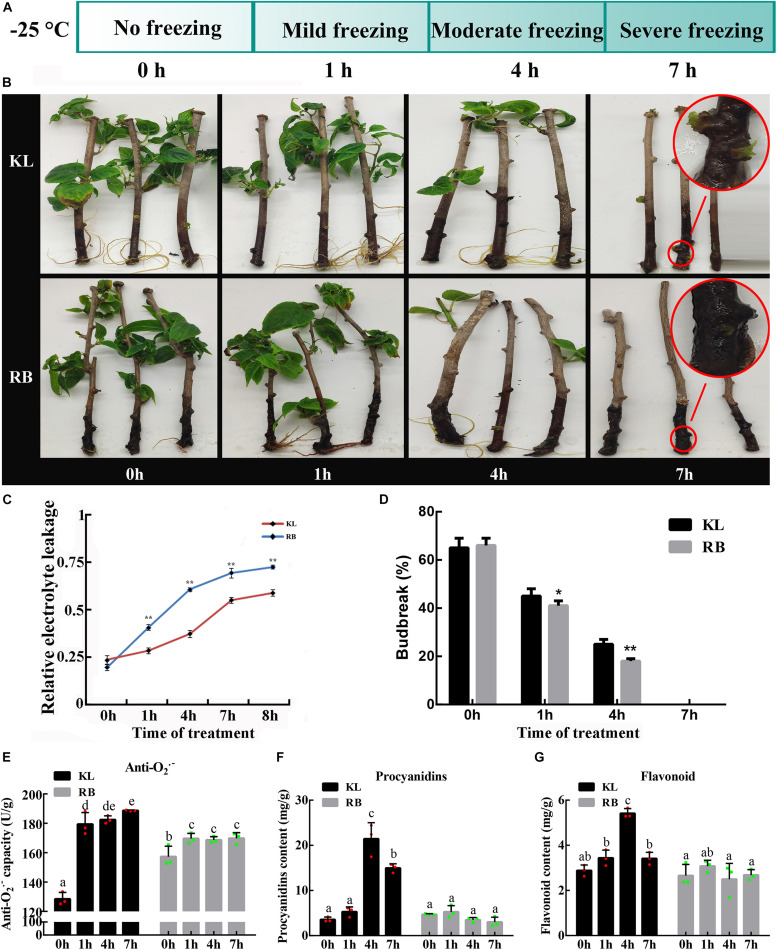
Different REL (relative electrolyte leakage) and VB (vegetative bud) between the KL and RB genotypes under the −25°C treatment. **(A)** Metabolomic and transcriptomic analysis of both genotypes were performed at four time points: 0 h, 1 h, 4 h, 7 h. **(B)** Representative images of KL and RB at 0 h, 1 h, 4 h, 7 h. **(C)** The REL at different treatment time points under −25°C. VB rates after the −25°C treatment, given as the mean of three independent replicate experiments in both genotypes. **(D)** VB rates after the −25°C treatment, given as the mean of three independent replicate experiments in both genotypes. **(E)** The anti−O_2_^.–^ capacity at different treatment. **(F)** The content of procyanidins at different treatment. **(G)** The content of flavonoid under −25°C.

After the freezing treatment (−25°C), four time-points corresponding to no freezing (0 h), mild freezing (1 h), moderate freezing (4 h), and severe freezing (7 h) were chosen for relative electrolyte leakage (REL), vegetative budbreak (VB), anti−O_2_^.–^ capacity and the contents of procyanidin, flavonoid measurements (Figure 2A). The REL of both genotypes gradually increased with a longer duration of the treatment (Figure 2C); however, the REL of KL was continuously lower than that of RB throughout the treatment. At 4 h, the REL of KL was below 50% while that of RB was above 50%. The REL of both genotypes was > 50% after 7 h at −25°C.

The VB rate was also assessed at the same four time points for both genotypes. Under the no freezing treatment (0 h/−25°C), the budbreak rate of both genotypes was above 60% and similar. At the second collection time point (1 h/−25°C), KL had a significantly higher budbreak rate than RB (*P* < 0.05). For the third time point (4 h/−25°C), compared to KL, the budbreak rate significantly decreased significantly to 20% in RB (*P* < 0.01). At the last collection time (7 h/−25°C), there was no VB (i.e., rates = 0%) in both plant genotypes. However, the shoots of KL were not completely dead and did generate new calluses, whereas the shoots of RB had thoroughly decayed (Figures 2B,D).

The anti−O_2_^.–^, procyanidin, and flavonoid were measured to evaluate the ROS scavenging ability of kiwifruit. An increasing trend for anti−O_2_^.–^ capacity in kiwifruit genotypes was observed under cold stress, however the KL had significantly stronger ability to scavenge ROS (Figure 2E). Low temperature accelerated the accumulation of procyanidin, and flavonoid in kiwifruit. The content of procyanidin in KL increased 3−fold from 0 h to 4 h, whereas the content of procyanidin in RB showed no changes during cold stress (Figure 2F). The content of flavonoid in KL initially showed increased and finally decreased trend, while no changes was observed in RB under cold stress. These results indicated that the high FT genotype had the high ROS scavenging ability compared with the low FT genotype (Figure 2G). For the further investigation, the four time points were also used in the metabolomic and transcriptomic analysis.

### Overview of Metabolic Profiles

**FIGURE 3:**
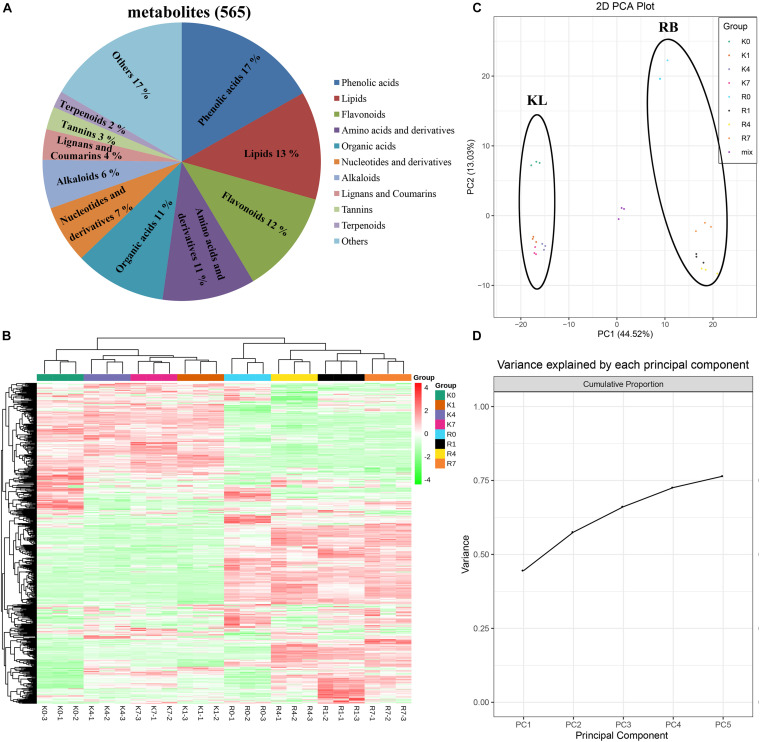
Overview and analysis of metabolites according to UPLC-ESI-MS/MS. **(A)** Classification of the 565 metabolites. **(B)** Clustering heat map of all metabolites. **(C,D)** PCA results. Score plot: the abscissa and the ordinate represented the scores of PC1 and PC2, respectively; the left ring represents KL, and the right ring represents RB. Cumulation plot: variance explained by PC1, PC2, PC3, PC4, and PC5.

We performed wide-targeted metabolic profiling based on UPLC-ESI-MS/MS. A total of 565 metabolites were identified among all the samples (Figure 3A), including phenolic acids (17%), lipids (13%), flavonoids (12%), amino acids, derivatives (11%), organic acids (11%), nucleotides and derivatives (7%), alkaloids (6%), lignans and coumarins (4%), tannins (3%), terpenoids (2%), and others (17%). In the heat map (Figure 3B), all the biological replicates were grouped together, which indicated a robust correlation between replicates and the high reliability of our data. The heat map also showed that some metabolites were specifically accumulated in the KL, yet some only existed in the RB. Further, disparate accumulation patterns of metabolites could be observed in both genotypes under cold stress, which implied that the intraspecific divergence of FT in *A. arguta* may be caused by differential metabolite contents. The clustering results of all samples showed that the time points clustered into two distinct groups (0 h vs. those at 1 h, 4 h, 7 h) in both KL and RB, which indicated their metabolic changes were consistent under low temperature stress. Moreover, after this treatment, the KL_4__–h_ and KL_1_,_7__–h_ were clustered into two categories and likewise RB_4__–h_ and R_1_,_7__–h_; this revealed that the 4 h (i.e., moderate freezing) duration is when specific metabolites changes occurred in both genotypes.

The data for the 565 metabolites were subjected to PCA analysis. The PCA score plot indicated that the first principal component (PC1) and the second principal component (PC2) represented 44.52% and 13.03% of the total variance, respectively (Figure 3C). The cumulative variance explained by PC1 to PC5 was above 75% (Figure 3D). Since the repeated samples and mixed samples were gathered tightly together, this indicated our experiment was reproducible and reliable. The two genotypes clearly separated along PC1 and PC2 under the normal condition without stress (0 h/−25°C) in the PCA score plot, indicating they had a distinguishable chemical composition under the no-freezing condition. For KL, its samples shifted to and clustered beneath of the no-freezing samples along PC2 after incurring cold stress. For RB, its samples showed the same pattern as KL but moved farther along PC2 than that in KL.

### Overview of Transcriptional Profiles

Twenty-four RNA libraries were built from RNA samples extracted from the two contrasting kiwifruit genotypes, KL (freezing-tolerant) and RB (freezing-sensitive), under the −25°C treatment applied. The Illumina sequencing of the 24 samples generated 171.39 Gb of clean reads. As shown in Supplementary Table 1, total mapped reads accounted for 63%−70% of the clean reads. Moreover, clean reads were assembled into 822 851 transcripts with a minimum length of 200 bp (Supplementary Figure 1a). Following a quality control analysis of the transcriptome assembly, 670 190 unigenes were conserved for further analyses. Unigenes could be annotated in the NR database, Nt database, KEGG Ortholog (KO), SwissProt, Pfam, Gene Ontology (GO), and eukaryotic Ortholog Groups (KOG) (Supplementary Figure 1b). The unigenes annotated in the NR database can convey the species distribution statistics (Supplementary Figure 1c), for which the top species was *Quercus suber*, with 60 311 transcripts, followed by *Vitis vinifera* (403,80, 10.99%). Annotation of the kiwifruit unigenes in the GO database classified 295 300 transcripts in the three main ontologies: biological processes, molecular functions, and cellular components (Supplementary Figure 1d). The terms having the largest numbers of transcripts were in the biological process category for which the most abundant terms were cellular process (181 963 transcripts) and metabolic process(160 737 transcripts), followed by biological regulation (73 475 transcripts) and response to stimulus (71 154 transcripts). For molecular function, the binding (178 271) and catalytic activity (156 437) processes were the most abundant terms found, while for cellular component, membrane change (104 070) was also a highly accumulated term.

In the KOG database, a total of 224 673 transcripts were annotated and divided 25 categories (Supplementary Figure 1e). Among those categories, only general function prediction had the largest number of transcripts (50 687), followed by post-translational modification, protein turnover, chaperones (247,41), with cell motility having the least number (72). In the classification of KOG annotations, carbohydrate transport and metabolism (12 554), amino acids transport and metabolism (9385), and lipid transport and metabolism (9457) were most pronounced, this result indicated that the sugar, amino acid, and lipid metabolism in *A. arguta* were relatively active, which could constitute the foundation for the healthy overwintering of *A. arguta*. The correlations tested between the samples replicates had Pearson | *r* | -values > 0.8, thus confirming the RNA-Seq data were repeatable and that our screening of differential genes by transcriptome data was reliable (Supplementary Figure 1f).

DEGs(differential expression genes) in both contrasting genotypes were screened. Comparison of the expression levels of DEGs between every groups were based on FPKM values. There were 1 856 DEGs between KL−0 h and KL−1 h, 2 632 DEGs between KL−0 h and KL−4 h, 1 708 DEGs between KL−0 h and KL−7 h, 4 011 DEGs between RB−0 h and RB−1 h, 4 309 DEGs between RB−0 h and RB−4 h, 2 798 DEGs between RB−0 h and RB−7 h (Supplementary Figure 2). These results indicated that DEGs were abundantly enriched at 4 h treatment in both genotypes.

### Comparison of Metabolite and Gene Levels Between KL and RB Under the No-Freezing Condition (0 h/−25°C)

**FIGURE 4:**
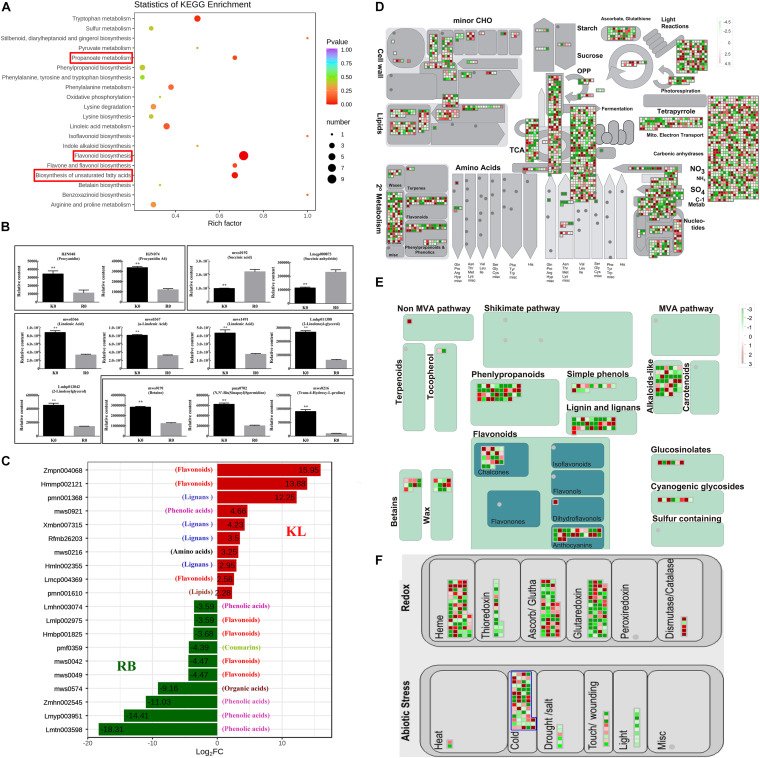
Comparison of the DEMs and DEGs between KL and RB under the no-freezing condition. **(A)** KEGG pathway enrichment analysis of DEMs. **(B)** DEMs were involved in flavonoid biosynthesis, propanoate metabolism and biosynthesis of unsaturated fatty acids. **(C)** Top 10 DEMs are shown for each genotype. **(D)** Overview of the pathways analyzed, via Mapman, in the DEGs of KL and RB under the no-freezing condition (0 h/−25°C). **(E)** DEGs involved in the secondary metabolism pathway. **(F)** DEGs involved in the redox metabolism pathway. These values are the medians from log2 (fold-change) values for each DEG. Red and green denote up-regulation and down-regulation, respectively.

Under the no-freezing condition (0 h/−25°C), the differentially expressed metabolites (DEMs) and genes (DEGs) between the two genotypes amounted to 157 and 40 452, respectively. KEGG enrichment results of DEMs showed that flavonoid biosynthesis, propanoate metabolism, and biosynthesis of unsaturated fatty acids differed between the two genotypes (Figure 4A). For flavonoid biosynthesis, both procyanidin (HJN074) and procyanidin-A6 (HJN048) had higher concentrations in KL than RB (Figure 4B). For propanoate metabolism, the succinic acid (mws0192) and succinic anhydride (Lmqp000873), involved in propanoate metabolism, RB had higher contents than did KL (Figure 4B). Propanoate metabolism, a component of the TCA cycle, plays a main role in diminishing tolerance to freezing. For the biosynthesis of unsaturated fatty acids, γ-linolenic acid (mws0366), α-linolenic acid (mws0367), linolenic acid (mws1491), 2-γ-linolenoyl-glycerol (Lmhp011388), and 2-linoleoylglycerol (Lmhp012042) in KL were higher than the corresponding amount detected in RB (Figure 4B). Other metabolites, namely betaine (mws0191), spermidine (pma07702), and proline (mws0216), were also found involved in FT to increase KL tolerance (Figure 4B). Compared with RB, the KL genotype had higher concentrations of flavonoid and lignan among the top 10 metabolites based on the fold-change between KL and RB. By contrast, RB accumulated higher contents of phenolic acid among the top 10 metabolites when compared with KL (Figure 4C). The total of 40 452 DEGs were identified in RB-0 h and KL-0 h, then the most of them were enriched in secondary metabolites (Supplementary Figures 3A,B). An overview of cellular metabolism was gleaned via the Mapman results for the DEGs; evidently, abundant DEGs between KL and RB were involved in the TCA cycle, nucleotides metabolism, and secondary metabolism (Figure 4D). These DEGs were enriched in the mechanism of flavonoids and betaines metabolism among secondary metabolism; though, more up-regulated DEGs were involved in KL with respect to flavonoid metabolism (Figure 4E). Other DEGs were found enriched in the cold response and redox metabolism. For the latter, more DEGs were identified for thioredoxin, ascorb/glutha, glutaredoxin, and dismutase/catalase (Figure 4F). Taking a combined view of the metabolome and transcriptome, the relative contents of DEMs including flavonoids, propanoate, lipids, betaines, and lignans were all consistent with the expression of DEGs involved in flavonoids, TCA, lipids, and betaines pathways.

### Comparison of Metabolite and Gene Levels Between KL and RB Under the Freezing Condition

**FIGURE 5:**
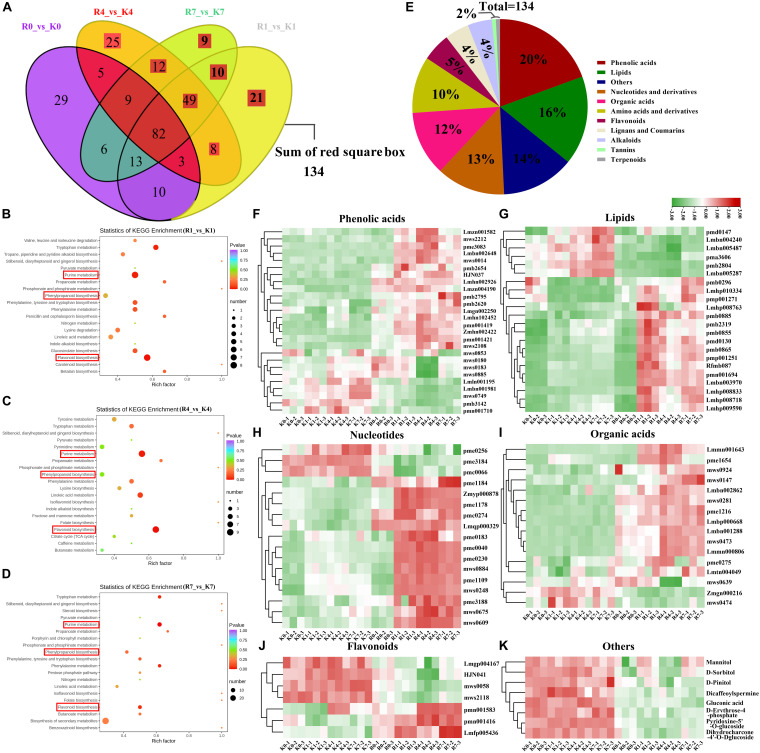
Comparison of the DEMs between KL and RB under the freezing condition. **(A)** Venn diagrams of DEMs of KL and RB at different treatment time points. **(B–D)** KEGG pathway enrichment of DEMs at different treatment time points. **(E)** Classification of the 134 specific under cold stress. **(F–K)** Heat map of metabolite responsive to cold in both KL and RB.

**FIGURE 6:**
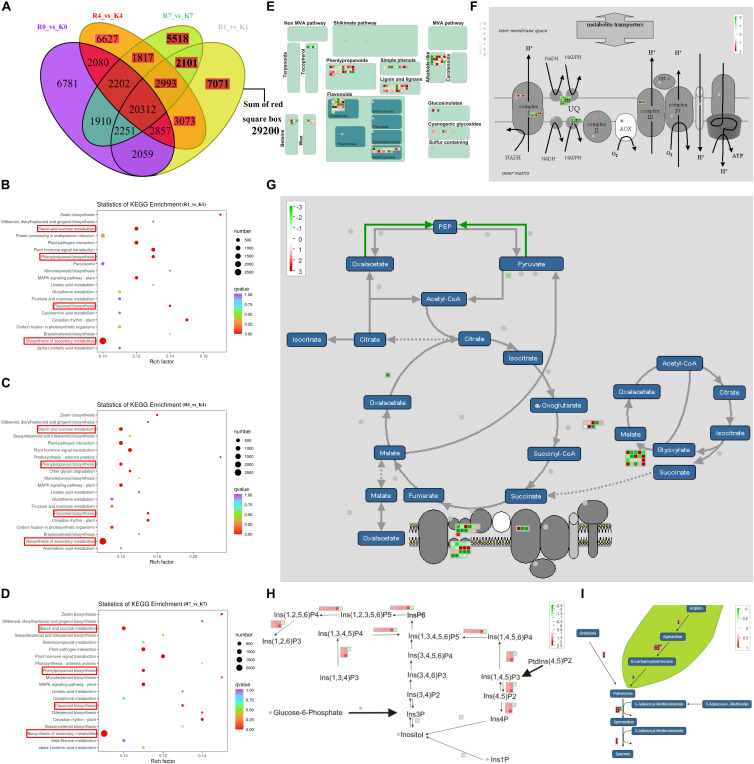
Comparison of the DEGs between KL and RB under the freezing condition. **(A)** Venn diagrams of DEGs of KL and RB at different treatment time points. **(B–D)** KEGG pathway enrichment of DEGs at different treatment time points. **(E–I)** The secondary metabolism, mitochondrial-electron-transport, TCA, inositol phosphates, spermine synthesis pathways, all analyzed via Mapman in DEGs. These values are the medians from log2(fpkm-KL/fpkm-RB) values of the 1 h, 4 h, and 7 h for each DEG. Red and green denote the up-regulation and down-regulation, respectively.

Comparison of the DEMs between the two genotypes revealed that there were 196, 193, and 190 DEMs at 1 h, 4 h, and 7 h, respectively (Figure 5A). The DEMs identified in the two genotypes under low temperature were analyzed using the KEGG pathway database. KEGG pathway analysis revealed that the most predominant subcategory among various pathways was ‘Purine metabolism’, followed by ‘Phenylpropanoid biosynthesis’, and ‘Flavonoid biosynthesis’ at 1 h, 4 h, and 7 h (Figures 5B–D). The 134 DEMs were specifically induced by low temperature, the classification of 134 DEMs showed that phenolic acids, lipids, nucleotides, organic acids, and flavonoids were the most abundant metabolites involved in the low temperature response of kiwifruit (Figure 5E). Most of the metabolites of phenolic acids, lipids, nucleotides, and organic acids were induced in RB compared with KL, however, most of the flavonoids were induced in KL compared with RB (Figures 5F–J). Moreover, the polysaccharide metabolites including Mannitol, Sorbitol, Pinitol, and Erythrose in KL had higher content than that in RB (Figure 5K). The antioxidant substance including erythrose-glucoside and dihydrochalcone-glucoside were specifically induced in KL. The osmotic substance dicaffeoylspermidine was induced in KL under low temperature, however, there was no changes in RB. To identify DEGs under cold stress in the two contrasting genotypes, comparison of the expression levels of DEGs between the two genotypes revealed that there were 42 717, 41 961, and 39 104 DEGs at 1 h, 4 h, and 7 h, respectively (Figure 6A). The DEMs identified in the two genotypes under low temperature were analyzed using the KEGG pathway database. KEGG pathway analysis revealed that the most predominant subcategory among various pathways was ‘Starch and sucrose metabolism’, followed by ‘Phenylpropanoid biosynthesis’, ‘Flavonoid biosynthesis’, and ‘Biosynthesis of secondary metabolites’ at 1 h, 4 h, and 7 h (Figures 6B–D). The 29 200 DEGs were specifically induced by low temperature, 29 200 DEGs were mapped in the pathways including secondary metabolism, mitochondrial-electron-transport, TCA, inositol phosphates, spermine synthesis (Figures 6E–I). The DEGs involved in Flavonoids and phenylpropanoids, inositol phosphates, and spermine synthesis pathways had higher expression in KL than that in RB. however, The DEGs involved in mitochondrial-electron-transport and TCA had higher expression in RB than that in KL.

### Commonly and Specifically Responding Metabolites in KL and RB Under the Cold Treatment

**FIGURE 7:**
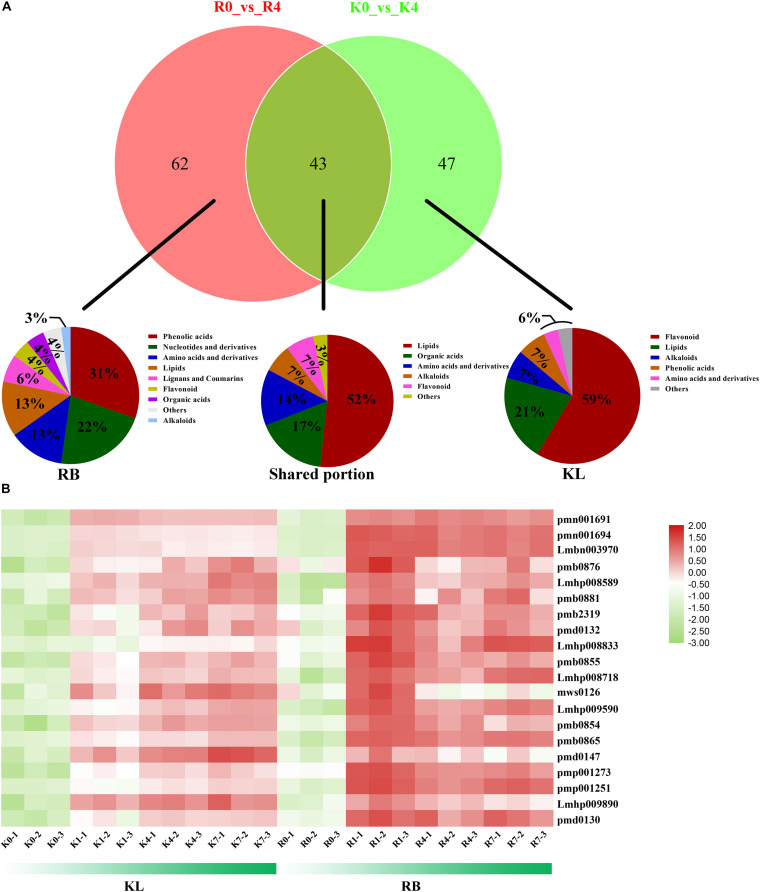
Comparison of metabolites levels and lipids between KL and RB genotypes under −4 h/−25°C treatment. **(A)** Venn diagrams of DEMs between both genotypes at the 4-h time point under −25°C. Classification of their common (shared) metabolites and of those accumulated only in KL and those only in RB. **(B)** Heat map of metabolite lipids responsive to cold in both KL and RB.

The Venn diagram showed that the DEMs common to KL and RB totaled 43 under cold stress, with most of the DEMs having a specific accumulation in both genotypes: 62 in RB and 47 in KL (Figure 7A). The classification of common DEMs showed that lipids were the metabolites most involved in the low temperature response of kiwifruit (Figure 7A). Those DEMs that only accumulated in KL were classified into five categories, for which flavonoid species were the most prevalent (Figure 7A). For the RB-specific DEMs, the classification results showed that phenolic acid mechanism and nucleotide metabolism were the most involved in the low temperature stress (Figure 7A). A total of 20 lipids (LysoPC 14:0, LysoPC 15:0, LysoPC 16:0, LysoPC 17:1, LysoPC 18:3, LysoPE 18:3, and so on) increased significantly in both genotypes after incurring cold stress when compared with their levels in the no-freezing condition (Figure 7B; Supplementary Table 2). With a longer exposure time to the freezing treatment, lipids was rapidly induced in first 1 h for RB and then gradually increased through the 4 h and 7 h. In contrast, the lipids accumulated continuously for KL under cold stress, but to lower level than that in RB.

**FIGURE 8:**
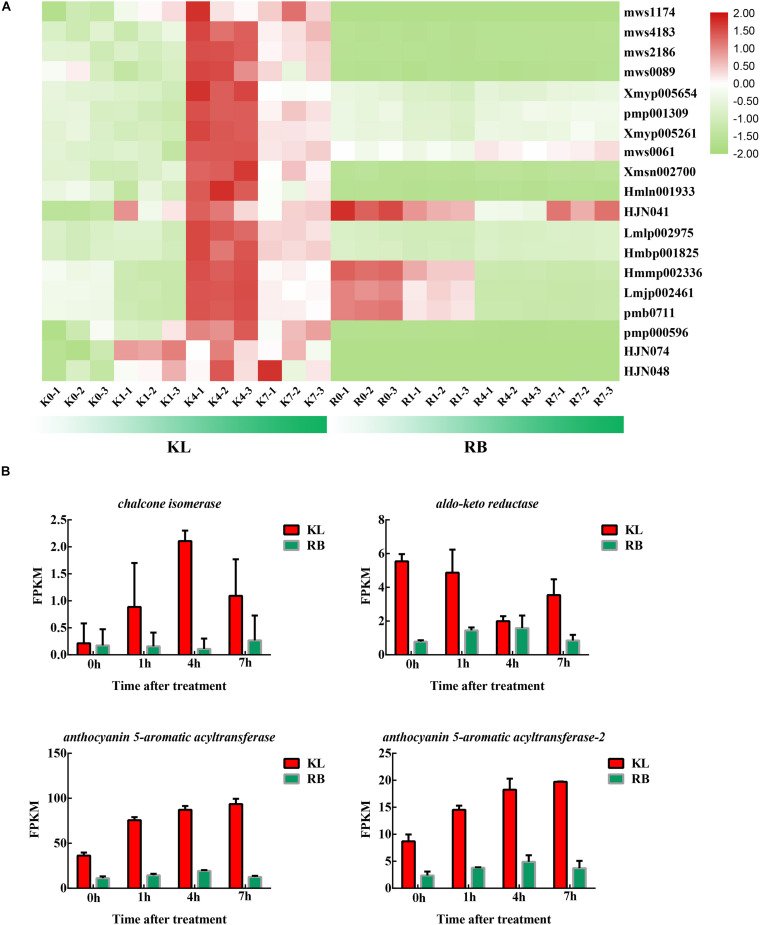
The DEMs and DEGs involved in the flavonoid metabolism pathway. **(A)** Heat map of flavonoids that specifically accumulated in the KL genotype. **(B)** Candidate DEGs involved in the flavonoid metabolism pathway.

Under cold stress, 19 flavonoids were upregulated remarkably in KL (Figure 8A; Supplementary Table 3), whereas flavonoids did not significantly change in RB. The flavonoids’ content in KL exceeded that in RB. Two flavonoids (HJN074, HJN048) were procyanidines, which could be induced by cold stress and have undergone specific accumulation in KL. The four DEGs involved in the flavonoid metabolism were induced by cold stress; moreover, 4 DEGs had higher expression levels in KL than in RB (Figure 8B).

**FIGURE 9:**
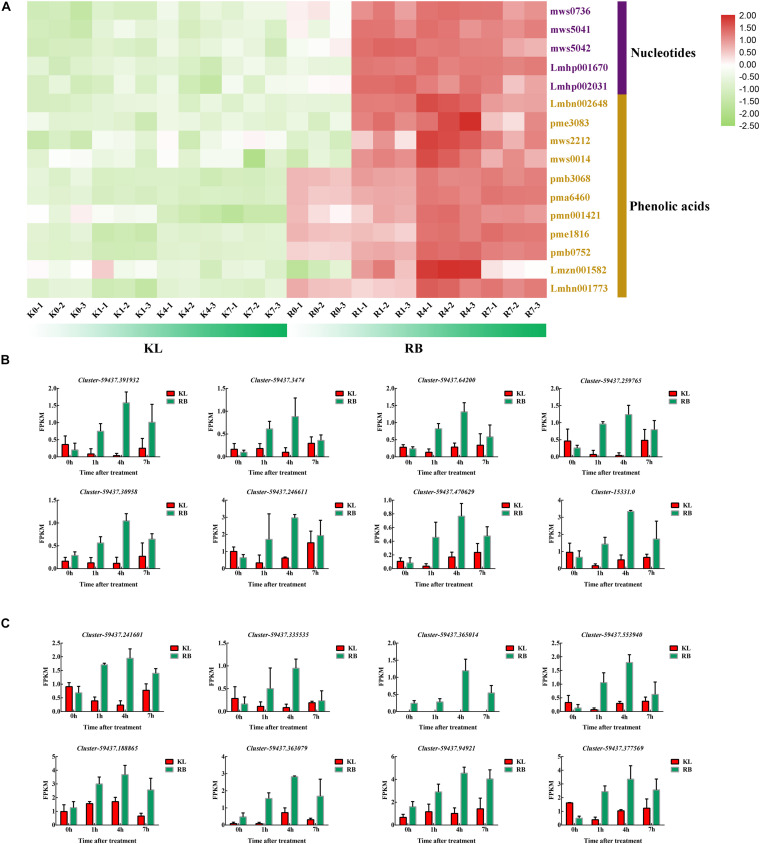
The DEMs and DEGs involved in the phenolic acids mechanism and nucleotides metabolism pathways. **(A)** Heat map of phenolic acids and nucleotides that specifically accumulated in the KL genotype. **(B)** Candidate DEGs involved in the nucleotides metabolism pathway. **(C)** Candidate DEGs involved in phenolic acids metabolism pathway.

The two types of metabolites, namely five nucleotides and 11 phenolic acids, increased significantly only in RB under cold stress (Figure 9A). The nucleotides’ metabolite level in RB was consistently higher than that in KL but also increased with longer exposure time to the low temperature treatment (Supplementary Table 4). The metabolic level of phenolic acid was similar to that of nucleotides; this was also moderately maintained at a high level in RB (Supplementary Table 5). In KL, however, it did not respond to the low temperature imposed. Eight DEGs were involved in nucleotides metabolism, and their gene expression levels were similar to those of metabolites (Figure 9B), being higher in RB than KL. Those eight DEGs are involved in phenolic acid metabolism, and their expression levels were also positively correlated with phenolic acid content (Figure 9C). The expression levels in RB significantly surpassed those in KL and displayed a trend of induction under prolonged low temperature exposure (Supplementary Table 6).

## Discussion

For perennial woody plants in temperate regions, the annual growth cycle can be divided into a growth stage (chilling tolerance) and dormancy stage (freezing tolerance, FT) (Owens et al., 1977). There is huge difference, however, in the tolerance mechanism at work between the growth and dormancy stages. It is therefore essential to separate these two stages when studying their molecular mechanisms for how plants respond to low temperature. In addition, to date, many previous studies have focused on the chilling tolerance (growth stage) of plants exposed to low temperatures, whereas their freezing tolerance in dormancy stage has received comparatively little attention.

The *A. arguta* species is widely distributed kiwifruit plant, and it has adapted to a broad range of environmental temperatures. The geographical distribution of KL and RB indicates that substantial divergence in their FT. The latter was quantified by the semi-lethal temperature (LT50), which confirmed this trait divergence, which amount to a 6°C between the genotypes. However, the LT50 of KL was lower than expected, perhaps because KL was planted in Henan, where the winter temperatures might have been insufficient for KL to reach its maximum FT. ROS scavenging ability is an important indicator of FT, KL had higher capacity to scavenge −O_2_^.–^ and accumulate higher contents of procyanidin and flavonoid compared with RB, which indicated ROS scavenging ability was the key factor to distinguish differences in FT. In this study, the REL and VB rates were also useful for evaluating the FT. Prior exposure to a −25°C, this temperature can stimulate the tolerance of KL and cause damage to RB, which could distinguish the differential FT of both genotypes. Moreover, varying the duration of this low temperature treatment let us better discern the FT in the both genotypes, which provided physiological evidence for our further omics experiment. In PCA results of 565 metabolites (Figure 3C), under cold stress, RB was positioned farther along PC2 than was KL when compared with untreated samples (0h at −25°C), which indicated the metabolite content of RB had a greater variation range under cold stress compared with KL. Based on above results, we may speculate that RB was sensitive to cold stress and strongly responded to damage from cold.

### Roles of Different Metabolism in the Kiwifruit Plant Response to Cold Stress

Both genotypes were cold acclimated to a maximum FT through the natural cooling of the field during the winter before treating them at −25°C. We uncovered differences in flavonoid metabolism, unsaturated fatty metabolism and TCA cycle between KL and RB under the no-freezing condition (0 h at −25°C). Flavonoid metabolites, acting as antioxidants to remove ROS, can increase FT (Li et al., 2019), while unsaturated fatty metabolism, by enhancing the cell membrane fluidity, can increase plant resistance to cold (Li et al., 2020). The TCA cycle represents energy metabolism, while energy metabolism meant that plant dormancy was weak (Wang et al., 2021). Betaine, arginine and proline can be used as osmotic stress substances to improve plants’ FT (Meilong et al., 2020). We found four lignans in KL that had higher concentrations than in RB. Lignans are known to have the ability to scavenge ROS and resist oxidation stress (Khan et al., 2020). In conclusion, osmotic ability and ROS scavenging ability were major factors driving the different FT of the kiwifruit genotypes. In the process of both genotypes responding to low temperature stress, differential metabolites were enriched in flavonoids metabolism and phenylpropanoid biosynthesis metabolism pathways; in this respect, flavonoids metabolism can improve plant cold resistance, while phenylpropanoid biosynthesis metabolism, especially phenolic acid metabolism, and TCA mechanism can also decrease plants’ FT to the cold (Huang et al., 2015; Thalmann and Santelia, 2017). Concerning polysaccharide and spermine, both can promote osmotic ability and make cold stress response more effective (Sebastián et al., 2018). For RB’s response under low temperature stress, both lipid metabolism and energy metabolism were more induced in RB than those in KL, which meant that RB’s energy metabolism remained operational in the freezing cold. While high-energy metabolism confers weak dormancy, leaving plants comprised in their ability to withstand injury from low temperatures.

### Lipid Metabolism as a Biomarker of Lipid Damages in the Plant Response to Cold Stress

Under cold stress, a contrasting FT was detected between KL (tolerant) and RB (sensitive) using the LC-MS/MS and RNA-Seq analyses. The compounds accumulated in both genotypes under cold stress represented fundamental metabolites responsive to cold stress. These metabolites, including various secondary metabolites, are known to contribute to cold stress tolerance and some of them metabolites were known to accumulate in other plant species (Colinet et al., 2012). Changes in these metabolites suggested conserved reconstruction in metabolome levels in plants in response to cold stress. In this study, the plasma membrane had crucial roles in the response to low temperature stress, as lipids are the major component of the plasma membrane. From our the metabolism analysis, we find that LPCs, LPEs, and free fatty acids were accumulated in both genotypes. Interestingly, three free fatty acids (pmn001691, pmn001694, Lmbn003970) all accumulated in RB more than in KL. The free fatty acids are the principal toxins of plant membrane lipid systems; while three LPEs, including LPE 16:0 (pmb0876), LPE 18:3 (Lmhp008589), and LPE 18:2 (pmb0881), were significantly accumulated only in RB under mild freezing (1 h at −25°C), then, these changes were becoming disordered. However, LPEs gradually increased in the KL genotype, which indicated that cold stress caused much more damage to the RB genotype. The LPCs’ results were the same as those found for LPEs. These suggested that lipid metabolism figures prominently in how kiwifruit responds to cold stress. In particular, the LPE 18:0 (2n isomer) (Tri760) was associated with phospholipids, and studies have shown it to be an isomer of lysophosphatidylethanolamine, which is a component of phospholipids and related to biofilm synthesis; moreover, cold resistance in plants is closely related to the fluidity of biofilms. LPCs can be produced by the non-enzymatic oxidation of phospholipids; LPCs are also produced by phospholipase A2 (PLA2), which cleaves phospholipids into LPC and fatty acids with specificity on the sn−2 position. Lipoxygenase (LOX) was found to be a key enzyme involved in lipid-signaling, adding oxygen to linoleic and linolenic acids to produce highly reactive lipid hydroperoxides (Alferez et al., 2010). The rapid accumulation of LPC and LPE has been reported in many plant species that incur by stress (Narvaez-Vasquez et al., 1999). Additionally, in research on blackberry red drupelet disorder research, phospholipids were a major constituent of cell membranes and the increased LPC level in red drupelet disorder indicated a loss of cell membrane integrity (Kim et al., 2019). Therefore, a higher LPC content may be an important biomarker for cold damage to plants.

### Induction of Flavonoid Metabolism Specifically in KL in Response to Cold Stress

A subset of metabolites specifically accumulated in KL. Genes involved in secondary metabolism, including those for flavonoid biosynthesis as well as flavone and flavonol biosynthesis, may have critical functions in a plant’s response to cold stress (Wei et al., 2019). For the flavonoid biosynthesis process, quercetin glycoside levels were dramatically increased (more than 10-fold) in the KL whereas the changes in RB were negligible. Quercetin glycosides showed a high ROS-scavenging activity. In addition, quercetin-3-O-arabinoside, quercetin-3-O-glucoside, quercetin-3-O-xylosyl-galactoside, quercetin-3-O-sambubioside, quercetin-O-feruloyl-pentoside, quercetin-3-O-neohesperidoside, quercetin-7-O-rutinoside, and quercetin-3,7-O-diglucoside all featured different antioxidant activities. Kaempferol glycosides also have an ROS-scavenging activity, and we found that kaempferol-7-O-glucoside, kaempferol-4′-O-glucoside, 6-hydroxykaempferol-7-O-glucoside were increased in KL. Therefore, perhaps the high quercetin and kaempferol contents in this more tolerant plant under the cold treatment contributed to higher antioxidant activity compared with RB. The increased accumulation of these compounds might be due to the upregulated expression of flavonoid biosynthesis genes. Anthocyanins are type of flavonoid compound that have crucial roles in plant survival by protecting cells against damage caused by various stress conditions (Xie and Chen, 2018). Anthocyanin biosynthesis was also affected by low temperatures, which influenced the expression of genes for flavonoid biosynthesis. The genes involved in anthocyanin biosynthesis were found affected by cold in this study, in that the genes of Cluster−59437.264203 (codeinone reductase), Cluster−59437.286871 (chalcone isomerase), Cluster−59437.250404, and Cluster−59437.316559 (anthocyanin 5−aromatic acyltransferase) were all upregulated in the KL. These results suggested that an increase in the expression level of a specific gene involved in the anthocyanin pathway would probably result in anthocyanin accumulation; they also indicated that anthocyanins could enhance the FT of kiwifruit. In grapevine (*Vitis*), it was reported that the loss of anthocyanin in grape berries at high temperatures arose from non-enzymatic degradation and inhibition of transcription in anthocyanin biosynthetic genes (Mori et al., 2007; Schulz et al., 2015). Taken together, we reasonably inferred that the mechanisms of anthocyanin synthesis in kiwifruit plants differ under low-temperature stresses, and this aspect of the FT trait merits further researched.

### Induction of Phenolic Acid and Nucleotides Metabolism, Particularly in RB, in Response to Cold Stress

One of the simplest ways to assess cold injuries in plant tissues is by observing the typical browning associated with membrane rupture. As compartmentalized phenolic compounds and enzymes are simultaneously released into the cell, the phenolics lose their esterified moieties and are then free to react with proteins, forming insoluble brown complexes (Chalker-Scott and Fuchigami, 1989). In this respect, α-hydroxycinnamic acid, 2−(formylamino)benzoic acid, caffeic acid, coniferyl alcohol, ferulic acid, 1−O-p-coumaroylquinic acid, 4−O-p-coumaroylquinic acid, 3−O-(E)-p-Coumaroylquinic acid, caffeoylquinic acid, 3−O-feruloylquinic acid, 5′-glucosyloxyjasmanic acid, caffeoylnicotinoyltartaric acid and syringoylcaffeoylquinic acid O-glucose were all found increased in the RB genotype after it incurred cold stress. Phenolic acids may be useful as an index damage index to gauge and evaluate cold damage to plants (Figure 9A). In addition, since ferulic acids and caffeic acids are downstream metabolites of TCA metabolism, this indicated that RB harbors a strong energy metabolism under cold stress conditions. Nucleotide metabolism could be attributed to cold-induced damage, and we found that adenine, 2−hydroxy-6−aminopurine, cytidine, adenosine, 2−hydroxyadenosine, guanosine, β-nicotinamide mononucleotide, guanosine monophosphate and cyclic AMP (adenosine monophosphate) were increased in RB exposed to cold. This cold stress caused damage to nucleotides damage that spurred an increase in nucleotide metabolism. Overall, eight genes involved in phenolic acids metabolism, plus another eight genes involved in nucleotides metabolism, for a total of 16 gene were up-regulated under cold stress conditions in RB, with no changes detected in KL. These differences may point to key metabolism features that have caused the divergence in FT between the KL and RB genotypes.

## Conclusion

**FIGURE 10:**
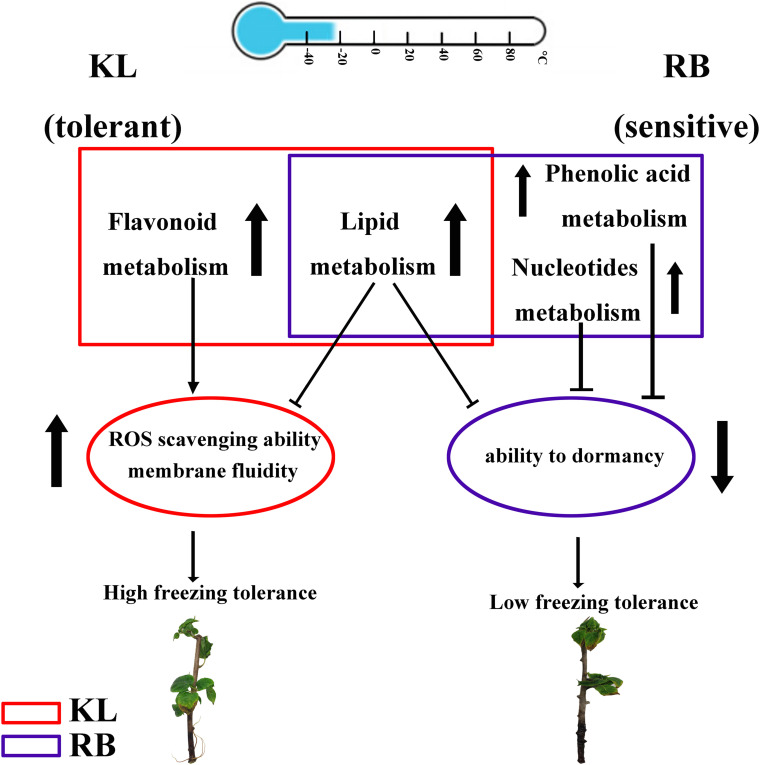
Proposed freezing tolerance models for *A. arguta*. In KL, flavonoid metabolism was specifically upregulated in KL under the cold treatment, which increased its ability to scavenge ROS. In RB, both phenolic acid metabolism and nucleotide metabolism were specifically upregulated in RB under the cold treatment, which decreased its ability to tolerate freezing by weakening its dormancy ability. For lipid metabolism, metabolites were accumulated in both genotypes, which impaired membrane integrity and could serve as biomarkers of cold damage to plants.

In this study, both the DEGs and DEMs in two kiwifruit genotypes differing in FT (freezing tolerance) were identified by metabolome and transcriptome analyses after imposing a cold stress treatment. In addition, unique metabolites and genes were detected in each genotype. Although lipid metabolites were common between KL and RB, they were significantly higher in RB and might be linked to cell membrane integrity and cold damage. Therefore, lipids could serve as a damage index to evaluate FT. Flavonoid metabolism, induced in KL only, could be involved in scavenging for ROS to enhance FT trait in KL. Both phenolic acid metabolism and nucleotide metabolism were induced in RB only, decreasing its FT. This comprehensive study thus provides new evidence and insights into how differing genotypes of the same species respond to cold stress at metabolomic and transcriptomic levels (Figure 10).

## Data Availability Statement

The datasets presented in this study can be found in online repositories. The names of the repository/repositories and accession number(s) can be found below: https://www.ncbi.nlm.nih.gov/, PRJNA681641.

## Author Contributions

SS, ML, XQ, JC, YZ, AM, ZL, and YL conducted the experiments. JF organized and supervised the overall project. SS and ML performed the data analysis and wrote the manuscript. CH edited the manuscript. All authors contributed to the article and approved the submitted version.

## Conflict of Interest

The authors declare that the research was conducted in the absence of any commercial or financial relationships that could be construed as a potential conflict of interest.

## Footnotes

1 www.r-project.org

2 http://www.kegg.jp/kegg/compound

3 http://www.kegg.jp/kegg/pathway.html

## Supplementary Material

Supplementary Figure 1 Overview and analysis of the transcriptome. (**a**) Distribution of transcripts sequence length. (**b**) Unigenes were annotated in 7 databases. (**c**) Unigenes were annotated in Nr database and species distribution statistics. (**d**) Unigenes were annotated in GO and classified into Biological process, Cellular component and Molecular Function. (**e**) Unigenes were annotated in KOG and classified into different function. (**f**) Pearson’s correlation coefficient analysis in different samples.

Supplementary Figure 2 Summary of differential expression analysis of kiwifruit under cold stress.

Supplementary Figure 3 Overview of DEGs in RB-0 h VS. KL-0 h. (**a**) The volcano plot between the RB-0 h and KL-0 h. (**b**) Enrichment of DEGs in the KEGG pathway.

Supplementary Table 1 The statistical results of clean reads mapped into reference.

Supplementary Table 2 Common responding metabolites lipids in KL and RB.

Supplementary Table 3 Flavonoids that specifically accumulated in KL.

Supplementary Table 4 Phenolic acids specific accumulated in RB.

Supplementary Table 5 Nucleotides specific accumulated in RB.

Supplementary Table 6 Candidate DEGs involved in flavonoid metabolism pathway, phenolic acid metabolism pathway and nucleotide metabolism pathway.
