# Supplementary figures and images for: Comparative Metabolomic and Transcriptomic Studies Reveal Key Metabolism Pathways Contributing to Freezing Tolerance Under Cold Stress in Kiwifruit
Source: Front Plant Sci. 2021 Jun 1;12:628969. doi: 10.3389/fpls.2021.628969 (PMC8204810; doi:10.3389/fpls.2021.628969)

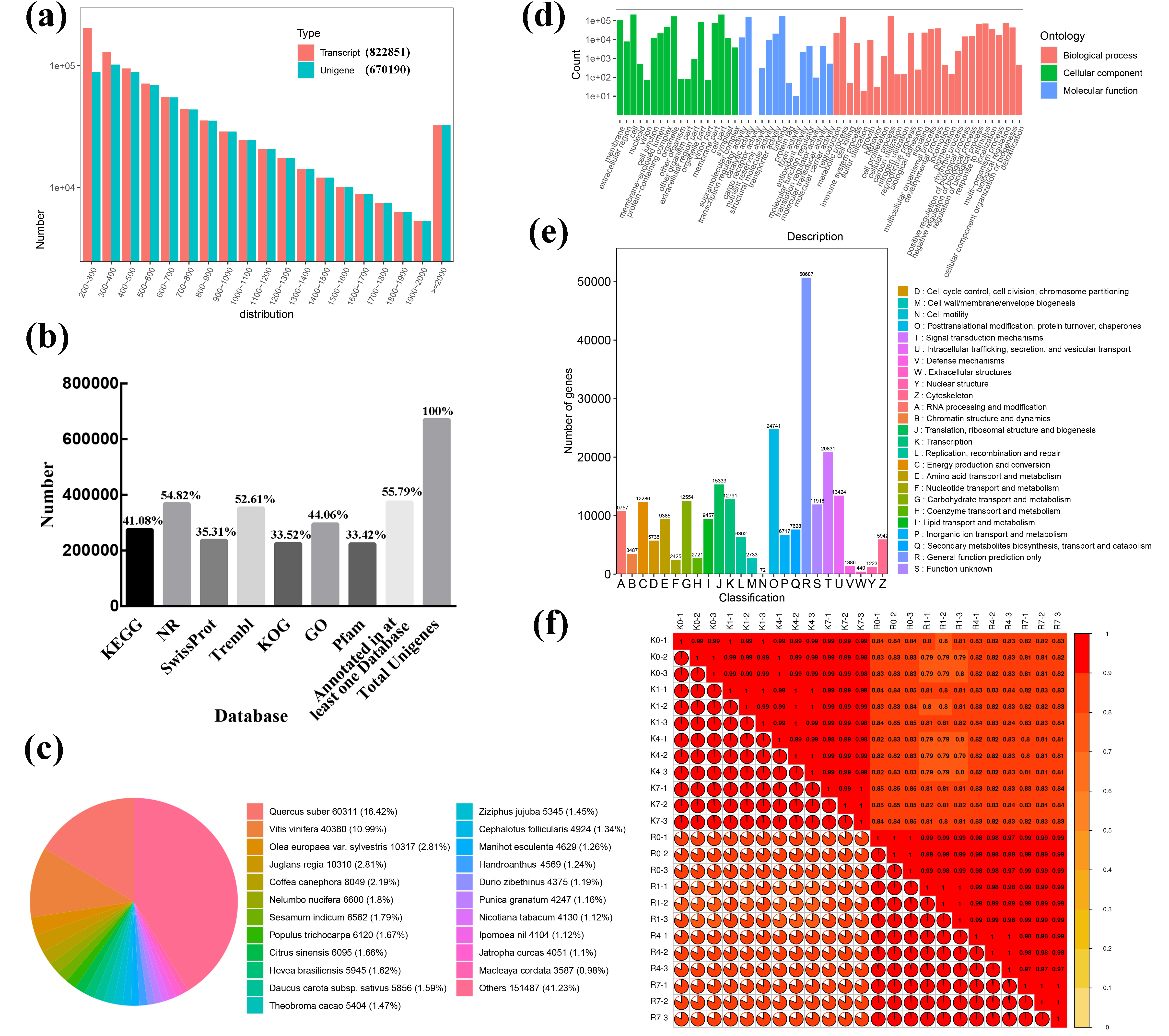

Supplement: Supplementary Figure 1 — Overview and analysis of the transcriptome. (a) Distribution of transcripts sequence length. (b) Unigenes were annotated in 7 databases. (c) Unigenes were annotated in Nr database and species distribution statistics. (d) Unigenes were annotated in GO and classified into Biological process, Cellular component and Molecular Function. (e) Unigenes were annotated in KOG and classified into different function. (f) Pearson’s correlation coefficient analysis in different samples. [file Image_1.jpg]

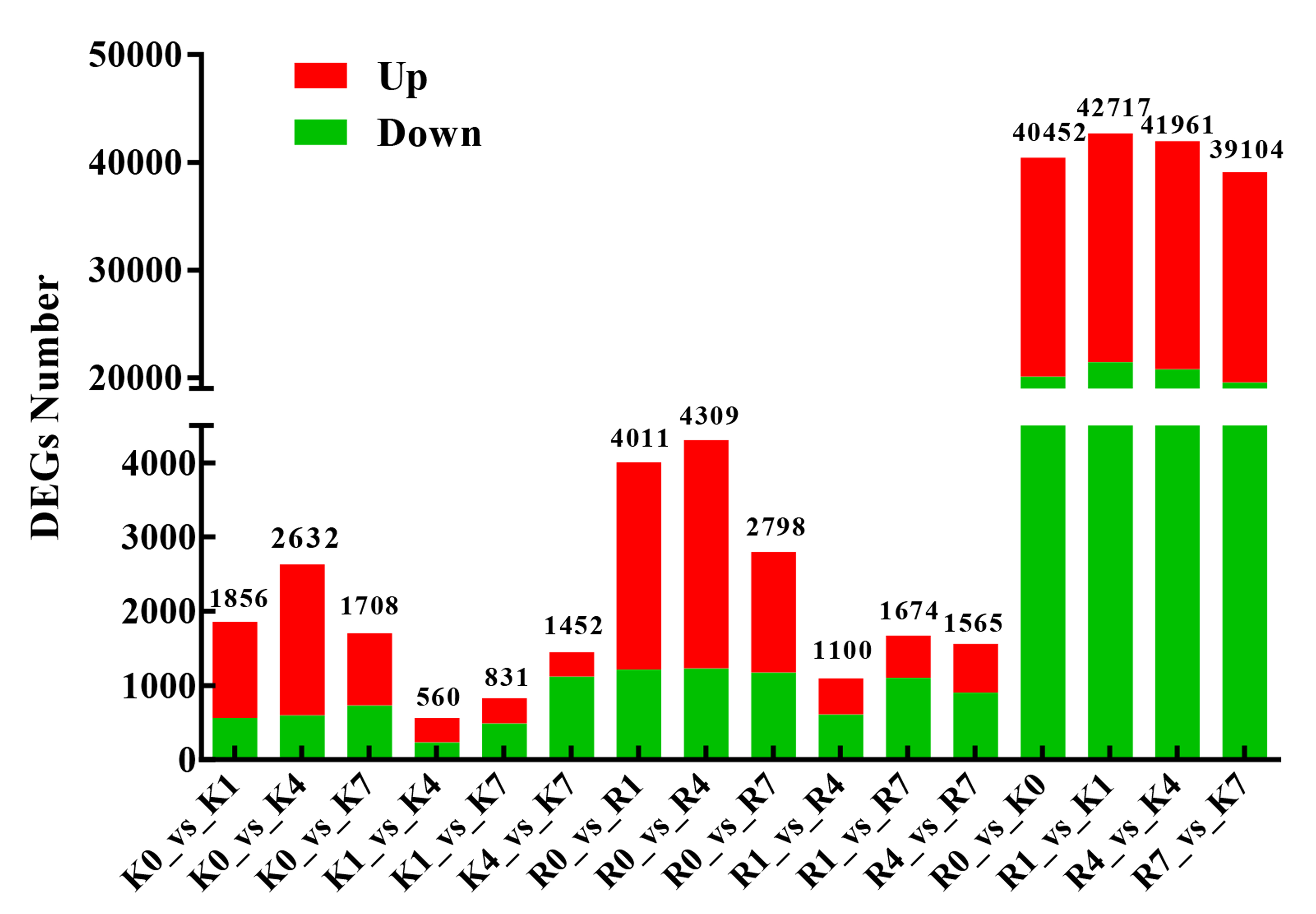

Supplement: Supplementary Figure 2 — Summary of differential expression analysis of kiwifruit under cold stress. [file Image_2.jpg]

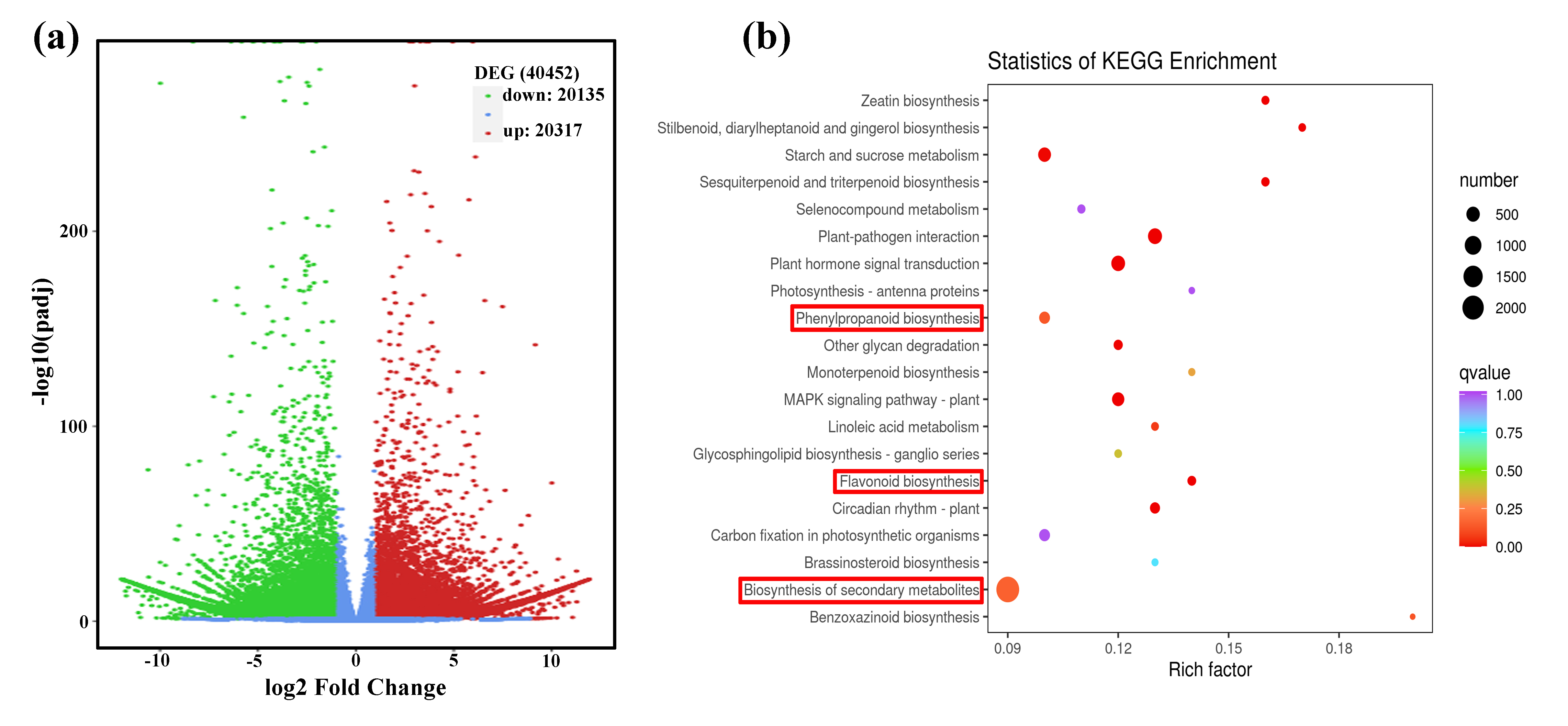

Supplement: Supplementary Figure 3 — Overview of DEGs in RB-0 h VS. KL-0 h. (a) The volcano plot between the RB-0 h and KL-0 h. (b) Enrichment of DEGs in the KEGG pathway. [file Image_3.jpg]
