# Supplementary material for: Comparative Metabolomic and Transcriptomic Studies Reveal Key Metabolism Pathways Contributing to Freezing Tolerance Under Cold Stress in Kiwifruit
Source: Front Plant Sci. 2021 Jun 1;12:628969. doi: 10.3389/fpls.2021.628969 (PMC8204810; doi:10.3389/fpls.2021.628969)
Supplement: Supplementary Table 1 — The statistical results of clean reads mapped into reference. [file Table_1.DOCX]

| **Sample** | **Clean Reads** | **Mapped Reads** | **Percentage** |
| --- | --- | --- | --- |
| K0-1 | 50618486 | 33656258 | 66% |
| K0-2 | 49599460 | 34480846 | 70% |
| K0-3 | 49385318 | 33938334 | 69% |
| K1-1 | 44465214 | 29373414 | 66% |
| K1-2 | 41588188 | 27850018 | 67% |
| K1-3 | 49820924 | 34037200 | 68% |
| K4-1 | 48836436 | 33614016 | 69% |
| K4-2 | 49122332 | 33431312 | 68% |
| K4-3 | 49400188 | 33207018 | 67% |
| K7-1 | 44610134 | 29741522 | 67% |
| K7-2 | 49026996 | 30918010 | 63% |
| K7-3 | 50403806 | 31892458 | 63% |
| R0-1 | 49490696 | 32830852 | 66% |
| R0-2 | 45530228 | 30616178 | 67% |
| R0-3 | 51109368 | 35003956 | 68% |
| R1-1 | 48917994 | 33397092 | 68% |
| R1-2 | 51623388 | 35337228 | 68% |
| R1-3 | 47057760 | 31253758 | 66% |
| R4-1 | 48794910 | 32226742 | 66% |
| R4-2 | 41314836 | 27428514 | 66% |
| R4-3 | 49988750 | 33226230 | 66% |
| R7-1 | 42113988 | 28070766 | 67% |
| R7-2 | 45576550 | 29067938 | 64% |
| R7-3 | 44149644 | 28644552 | 65% |

Table S1 The statistical results of clean reads mapped into reference
