# Supplementary material for: Comparative Metabolomic and Transcriptomic Studies Reveal Key Metabolism Pathways Contributing to Freezing Tolerance Under Cold Stress in Kiwifruit
Source: Front Plant Sci. 2021 Jun 1;12:628969. doi: 10.3389/fpls.2021.628969 (PMC8204810; doi:10.3389/fpls.2021.628969)
Supplement: Supplementary Table 2 — Common responding metabolites lipids in KL and RB. [file Table_2.DOC]

Table S2 Common responding metabolites lipids in KL and RB

| Index | Compounds | Class |
| --- | --- | --- |
| pmn001691 | Trihyroxy-octadecadienoic acid | Free fatty acids |
| pmn001694 | Trihyroxy-octadecadienoic acid | Free fatty acids |
| pmd0130 | LysoPC 14:0 | LPC |
| Lmhp008589 | LysoPE 18:3 | LPE |
| pmb0881 | LysoPE 18:2 | LPE |
| pmb2319 | LysoPC 15:0 | LPC |
| Lmhp008833 | LysoPC 16:1 | LPC |
| pmb0855 | LysoPC 16:0 | LPC |
| Lmhp008718 | LysoPC 17:2 | LPC |
| Lmhp009590 | LysoPC 17:1 | LPC |
| pmb0854 | LysoPC 18:3 | LPC |
| pmb0865 | LysoPC 18:3(2n isomer) | LPC |
| pmp001273 | LysoPC 18:2 | LPC |
| pmp001251 | LysoPC 18:2(isomer) | LPC |
| Lmhp009890 | LysoPC 20:3 | LPC |
| Lmbn003970 | Trihydroxy-octadecenoic acid | Free fatty acids |
| pmb0876 | LysoPE 16:0 | LPE |
| pmd0132 | LysoPC 16:0(2n isomer) | LPC |
| mws0126 | LysoPC 18:0 | LPC |
| pmd0147 | LysoPC 20:2 | LPC |
| pmp001276 | Dihydroxypropyl-octadecatrienoate-hexose | Free fatty acids |
