# Supplementary material for: Comparative Metabolomic and Transcriptomic Studies Reveal Key Metabolism Pathways Contributing to Freezing Tolerance Under Cold Stress in Kiwifruit
Source: Front Plant Sci. 2021 Jun 1;12:628969. doi: 10.3389/fpls.2021.628969 (PMC8204810; doi:10.3389/fpls.2021.628969)
Supplement: Supplementary Table 3 — Flavonoids that specifically accumulated in KL. [file Table_3.DOCX]

Table S3 Flavonoids that specifically accumulated in KL.

| Index | Compounds | Class |
| --- | --- | --- |
| mws1174 | 3-O-Acetylpinobanksin | Dihydroflavonol |
| mws4183 | Quercetin-3-O-arabinoside(guaijaverin)* | Flavonols |
| mws2186 | Avicularin* | Flavonols |
| mws0089 | Kaempferol-7-O-glucoside* | Flavonols |
| Xmyp005654 | Kaempferol-4'-O-glucoside* | Flavonoid |
| pmp001309 | 6-Hydroxykaempferol-7-O-glucoside* | Flavonols |
| Xmyp005261 | Quercetin-3-O-glucoside* | Flavonoid |
| mws0061 | Quercetin-3-O-Galactoside (Hyperoside)* | Flavonols |
| Xmsn002700 | Taxifolin-3'-O-glucoside | Flavonoid |
| Hmln001933 | Myricetin-3-O-galactoside* | Flavonols |
| HJN041 | Epicatechin glucoside | Flavonols |
| Lmlp002975 | Quercetin-3-O-xylosyl-(1→2)-galactoside* | Flavonoid |
| Hmbp001825 | Quercetin-3-O-sambubioside* | Flavonols |
| Hmmp002336 | Quercetin-O-feruloyl-Pentoside | Flavonols |
| Lmjp002461 | Quercetin-3-O-neohesperidoside* | Flavonols |
| pmb0711 | Quercetin-7-O-rutinoside* | Flavonols |
| pmp000596 | Quercetin-3,7-O-diglucoside | Flavonols |
| HJN074 | Procyanidin A6 | Flavonoid |
| HJN048 | Procyanidin | Flavonoid |
