# Supplementary material for: Comparative Metabolomic and Transcriptomic Studies Reveal Key Metabolism Pathways Contributing to Freezing Tolerance Under Cold Stress in Kiwifruit
Source: Front Plant Sci. 2021 Jun 1;12:628969. doi: 10.3389/fpls.2021.628969 (PMC8204810; doi:10.3389/fpls.2021.628969)
Supplement: Supplementary Table 4 — Phenolic acids specific accumulated in RB. [file Table_4.DOC]

| Index | Compounds | Class |
| --- | --- | --- |
| Lmbn002648 | α-Hydroxycinnamic Acid* | Phenolic acids |
| pme3083 | 2-(Formylamino)benzoic acid | Phenolic acids |
| mws2212 | Caffeic acid | Phenolic acids |
| mws0093 | Coniferyl alcohol | Phenolic acids |
| mws0014 | Ferulic acid | Phenolic acids |
| pmb3068 | 1-O-p-Coumaroylquinic acid* | Phenolic acids |
| pma6460 | 4-O-p-Coumaroylquinic acid* | Phenolic acids |
| pmn001421 | 3-O-(E)-p-Coumaroyl quinic acid* | Phenolic acids |
| pme1816 | Caffeoylquinic acid* | Phenolic acids |
| pmb0752 | 3-O-Feruloylquinic acid | Phenolic acids |
| Lmzn001582 | 5'-Glucosyloxyjasmanic acid | Phenolic acids |
| Lmhn001773 | Caffeoylnicotinoyltartaric acid | Phenolic acids |
| Lmgn002253 | Syringoylcaffeoylquinic acid O-glucose | Phenolic acids |

Table S4 Phenolic acids specific accumulated in RB
