# Supplementary material for: Comparative Metabolomic and Transcriptomic Studies Reveal Key Metabolism Pathways Contributing to Freezing Tolerance Under Cold Stress in Kiwifruit
Source: Front Plant Sci. 2021 Jun 1;12:628969. doi: 10.3389/fpls.2021.628969 (PMC8204810; doi:10.3389/fpls.2021.628969)
Supplement: Supplementary Table 5 — Nucleotides specific accumulated in RB. [file Table_5.DOC]

| Index | Compounds | Class |
| --- | --- | --- |
| pme0040 | Adenine | Nucleotides and derivatives |
| pme0183 | 2-Hydroxy-6-aminopurine | Nucleotides and derivatives |
| pme3732 | Cytidine | Nucleotides and derivatives |
| pme0230 | Adenosine* | Nucleotides and derivatives |
| Zmyp000878 | 2-Hydroxyadenosine | Nucleotides and derivatives |
| pme1178 | Guanosine | Nucleotides and derivatives |
| mws0675 | β-Nicotinamide mononucleotide | Nucleotides and derivatives |
| mws0609 | Guanosine monophosphate | Nucleotides and derivatives |
| mws0884 | Cyclic AMP | Nucleotides and derivatives |

Table S5 Nucleotides specific accumulated in RB
