# Supplementary material for: Comparative Metabolomic and Transcriptomic Studies Reveal Key Metabolism Pathways Contributing to Freezing Tolerance Under Cold Stress in Kiwifruit
Source: Front Plant Sci. 2021 Jun 1;12:628969. doi: 10.3389/fpls.2021.628969 (PMC8204810; doi:10.3389/fpls.2021.628969)
Supplement: Supplementary Table 6 — Candidate DEGs involved in flavonoid metabolism pathway, phenolic acid metabolism pathway and nucleotide metabolism pathway. [file Table_6.DOC]

| Category | ID | NR |
| --- | --- | --- |
| Flavonoid metabolism | Cluster-59437.264203 | codeinone reductase |
|  | Cluster-59437.286871 | chalcone isomerase |
|  | Cluster-59437.250404 | anthocyanin 5-aromatic acyltransferase |
|  | Cluster-59437.316559 | anthocyanin 5-aromatic acyltransferase |
| Phenoid acids metabolism | Cluster-59437.241601 | acyl-coa ligase easd |
|  | Cluster-59437.335535 | acyl-coa ligase azaf |
|  | Cluster-59437.365014 | trans-resveratrol di-O-methyltransferase-like |
|  | Cluster-59437.553940 | predicted protein |
|  | Cluster-59437.188865 | phenylalanine ammonia-lyase |
|  | Cluster-59437.363079 | phenylalanine ammonia-lyase |
|  | Cluster-59437.94921 | phenylalanine ammonia-lyase |
|  | Cluster-59437.377569 | cytochrome p450 monooxygenase psod |
| Nucleotides metabolism | Cluster-59437.391932 | amidophosphoribosyltransferase |
|  | Cluster-59437.3474 | bifunctional purine biosynthetic protein ade1 |
|  | Cluster-59437.64200 | bifunctional purine biosynthesis protein ade17 |
|  | Cluster-59437.259765 | inosine-5'-monophosphate dehydrogenase |
|  | Cluster-59437.30958 | gmp synthase |
|  | Cluster-59437.246611 | carbamoyl-phosphate synthase |
|  | Cluster-59437.470629 | ctp synthase |
|  | Cluster-15331.0 | adenosine kinase |

Table S6 Candidate DEGs involved in flavonoid metabolism pathway, phenolic acid metabolism pathway and nucleotide metabolism pathway.
