# Supplementary material for: Comparative Metabolomic and Transcriptomic Studies Reveal Key Metabolism Pathways Contributing to Freezing Tolerance Under Cold Stress in Kiwifruit
Source: Front Plant Sci. 2021 Jun 1;12:628969. doi: 10.3389/fpls.2021.628969 (PMC8204810; doi:10.3389/fpls.2021.628969)
Supplement: Supplementary file 10 [file Data_Sheet_1.doc]

**Figure S1** Overview and analysis of the transcriptome. (a) Distribution of transcripts sequence length. (b) Unigenes were annotated in 7 databases. (c) Unigenes were annotated in Nr database and species distribution statistics. (d). Unigenes were annotated in GO and classified into Biological process, Cellular component and Molecular Function. (e) Unigenes were annotated in KOG and classified into different function. (f). Pearson’s correlation coefficient analysis in different samples.

**Figure S2** Summary of differential expression analysis of kiwifruit under cold stress.

**Figure S3** Overview of DEGs in RB-0 h VS. KL-0 h. (a) The volcano plot between the RB-0 h and KL-0 h. (b) Enrichment of DEGs in the KEGG pathway.
